# Emerging Trends in the West Nile Virus Epidemiology in Croatia in the ‘One Health’ Context, 2011–2020

**DOI:** 10.3390/tropicalmed6030140

**Published:** 2021-07-24

**Authors:** Tatjana Vilibic-Cavlek, Vladimir Savic, Ana Klobucar, Thomas Ferenc, Maja Ilic, Maja Bogdanic, Irena Tabain, Vladimir Stevanovic, Marija Santini, Marcela Curman Posavec, Suncica Petrinic, Iva Benvin, Ivana Ferencak, Vlatko Rozac, Ljubo Barbic

**Affiliations:** 1 Department of Virology, Croatian Institute of Public Health, 10000 Zagreb, Croatia; maja.bogdanic11@gmail.com (M.B.); irena.tabain@hzjz.hr (I.T.); ivana.ferencak@hzjz.hr (I.F.); 2 Department of Microbiology, School of Medicine, University of Zagreb, 10000 Zagreb, Croatia; 3 Poultry Center, Croatian Veterinary Institute, 10000 Zagreb, Croatia; v_savic@veinst.hr; 4 Department of Epidemiology, Andrija Stampar Teaching Institute of Public Health, 10000 Zagreb, Croatia; ana.klobucar@stampar.hr (A.K.); marcela.curman@stampar.hr (M.C.P.); suncica.petrinic@stampar.hr (S.P.); 5 Clinical Department of Diagnostic and Interventional Radiology, Merkur University Hospital, 10000 Zagreb, Croatia; thomas.ferenc95@gmail.com; 6 Department of Epidemiology, Croatian Institute of Public Health, 10000 Zagreb, Croatia; maja.ilic@hzjz.hr; 7 Department of Microbiology and Infectious Diseases with Clinic, Faculty of Veterinary Medicine, University of Zagreb, 10000 Zagreb, Croatia; vladostevanovic@gmail.com (V.S.); ibenvin@vef.hr (I.B.); ljubo.barbic@vef.hr (L.B.); 8 Department for Intensive Care Medicine and Neuroinfectology, University Hospital for Infectious Diseases “Dr Fran Mihaljevic”, 10000 Zagreb, Croatia; marija.santini.ms@gmail.com; 9 Public Institution Nature Park Kopacki Rit, 31327 Kopacevo, Croatia; vlatko.rozac@pp-kopacki-rit.hr

**Keywords:** West Nile virus, epidemiology, ‘One Health’, Croatia

## Abstract

West Nile virus (WNV) is one of the most widely distributed (re-)emerging arboviruses. In Croatia, acute WNV infections as well as seropositivity were detected in humans, horses, birds and poultry. Although serologic evidence of WNV human infections dates back to the 1970s, no clinical cases were reported until 2012. WNV outbreaks, as well as sporadic infections, were continuously recorded in continental Croatian counties from 2012 to 2018. In addition, acute asymptomatic infections (IgM antibodies) in horses have been regularly notified in continental regions since 2012, while seropositive horses (seroprevalence rates 3.7–21.4%) were detected in both continental and coastal regions. Moreover, WNV seropositivity in poultry (1.8–22.9%) was reported from 2013 to 2020. During the largest WNV outbreak in 2018, WNV RNA was detected for the first time in two dead goshawks (*Accipiter gentilis*) from the same aviary in North-West Croatia, while WNV antibodies were found in one buzzard (*Butteo butteo*) from the same region. In addition, WNV RNA was detected in a dead blackbird (*Turdus merula*) at the Croatian littoral. The phylogenetic analysis of 11 strains detected in urine samples of patients with neuroinvasive disease and 1 strain detected in a goshawk showed circulation of WNV lineage 2. Thus far, WNV has not been detected in mosquitoes in Croatia.

## 1. Introduction

West Nile virus (WNV) is a widely distributed (re-)emerging arbovirus. Since its first descriptions in Uganda (1937), the virus has gradually dispersed, via migratory birds, out of Africa [1]. In Europe, large WNV outbreaks in humans and horses were reported in the 1990s. The outbreak that occurred in the area around Bucharest, Romania (1996) was the first WNV outbreak in a predominantly urban area associated with a large number of neuroinvasive cases [2]. Similarly, in the outbreak occurring in the Volgograd region, Russia (1999), the disease was more severe, with the central nervous system usually being involved and a higher case–fatality rate [3]. In horses, cases of WNV encephalitis were initially recorded in Italy (1998) and southern France (2000) [1]. Since 2010, WNV infections have been continuously reported in many European countries [4,5]. An unusually intense and early WNV transmission season was recorded in Europe in 2018. EU and EU-neighboring countries reported 2083 human cases and 180 deaths due to WNV [6,7]. The number of newly affected areas (*n* = 45) was higher than in previous years, suggesting a wider spread of WNV [7].

In nature, WNV is maintained in a bird–mosquito–bird transmission cycle and occasionally infects humans. The main vector of WNV in Europe is *Culex pipiens*, due to its vector competence, feeding preferences, and high abundance during summer; however, the virus was also found in other native *Culex* mosquito species (*Cx. modestus*, *Cx. perexiguus*) [8]. The experimental competence to transmit the WNV was proven for native (*Cx. torrentium*) and invasive mosquitoes (*Aedes albopictus*) [4]. Humans and horses represent incidental or ‘dead-end’ hosts for WNV. Additionally, WNV antibodies were detected in different animal species (cattle, sheep, goats, camels, deer, moose, squirrels) [9,10,11,12,13] as well as in pet animals (dogs, cats); however, clinical disease is rarely reported [14,15,16,17].

Although the majority of human WNV infections are asymptomatic (80%) or present as a self-limited febrile disease (WNV fever; 20%), some patients develop neuroinvasive infection (WNV neuroinvasive disease—WNND; meningitis, encephalitis, poliomyelitis) [18]. Risk factors associated with developing WNND include older age, a history of organ transplantation and possibly other immunosuppressive conditions [19,20]. Mortality in patients with WNND is 10% and the case–fatality ratio increases considerably with increasing age [21].

There are several genetic lineages, of which lineages 1 and 2 are associated with human diseases [22]. WNV lineage 1 was responsible for WNV outbreaks in the Mediterranean basin over the past 50 years [23]. WNV lineage 2 emerged in Europe in 2004 (Southeast Hungary) [24] and in subsequent years spread into neighboring countries [25]. WNV lineage 1 was associated with severe and fatal cases of WNV infection in humans, horses and avian species, suggesting that lineage 1 strains had increased pathogenicity, while lineage 2 strains were considered of low virulence [26]. However, repeated outbreaks of WNV neuroinvasive disease in Europe since 2010 caused by lineage 2 have demonstrated that both lineages have the potential for severe and fatal WNND [27].

In Croatia, seroepidemiological studies on WNV were conducted in the 1970s and 1980s [28,29]; however, there was no data on the WNV circulation thereafter. After the introduction of a passive flavivirus surveillance program in 2011 by the Croatian Ministry of Agriculture, Fisheries and Rural Development, seroprevalence studies in sentinel horses and poultry were continuously performed. Additionally, from 2017 to 2021, a project on the prevalence and molecular epidemiology of emerging and re-emerging neuroinvasive arboviral infections (CRONEUROARBO) was conducted. The project included the detection of arboviruses in humans, sentinel animals (horses, birds, poultry) and vectors (mosquitoes, ticks) in continental Croatian regions.

Thus far, WNV infections have been reported in humans (2012–2018), horses (2011–2020), birds (2018) and poultry (2013–2020) (Table 1).

## 2. WNV Infections in Humans

The first serologic evidence of WNV in humans dates back to the 1970s. Two studies conducted among residents of the Island Brač (Middle Dalmatia) showed the hemagglutination-inhibiting (HI) antibody seroprevalence of 4.9% (1970) and 0.28% (1974), respectively [28]. In a serosurvey conducted in 1980, WNV HI antibodies were found in 1.2% of inhabitants from northeastern Croatia, 3.4% from Middle Dalmatia and 0.8% of inhabitants from southern Dalmatia [29]. A similar neutralizing (NT) antibody prevalence rate of 0.3% was detected in voluntary blood donors from northeastern Croatia (2007) [30] and asymptomatic individuals aged 30–60 years from continental Croatia (2011) [31].

Clinical WNV cases were not recorded until 2012, when an outbreak (seven cases) of WNV neuroinvasive disease (WNND) occurred in three eastern Croatian counties [32]. In 2013, an outbreak involving 20 cases was reported in two northwestern counties [33]. Only sporadic infections were detected from 2014 to 2016. A small outbreak (eight cases) was recorded in 2017, followed by the largest outbreak so far in 2018, with 54 WNND and 7 WNV fever cases detected in 11 Croatian counties [34]. Areas affected in 2018 were those in which cases were also reported between 2012 and 2017 [32,33], while in three areas, human cases were reported for the first time [34]. No human clinical cases were notified in 2019 and 2020. WNV seropositive individuals were continuously recorded in counties where acute cases occurred [35]. Thus far, there are no reported WNV infections at the Croatian littoral.

WNV incidence rates in Croatia varied annually, from 0 to 1.49/100,000 population (crude incidence rate 0.24/100,000 population). Similar trends were observed in some neighboring countries. During the same period, annual incidence rates in Greece varied from 0/100,000 population in 2015 and 2016 to 2.2/100,000 population in 2018 [36]. In addition, notification rates in Italy were from 0.06 to 1.83 cases/100,000 inhabitants [37]. Like in Italy and Greece, the incidence rates remained relatively low; however, the persistent WNV circulation suggests that WNV is becoming endemic in Croatia [34,36,37].

**Figure 1:**
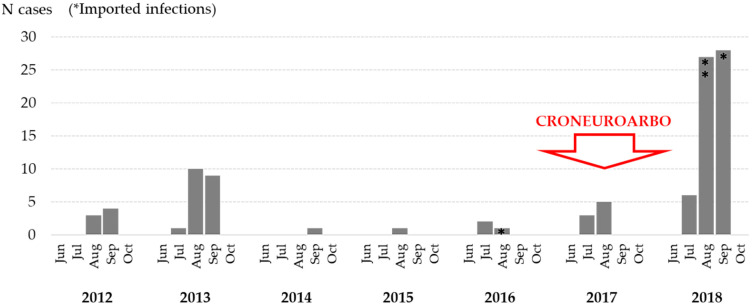
Seasonal distribution of human WNV infections in Croatia, 2012–2018.

**Figure 2:**
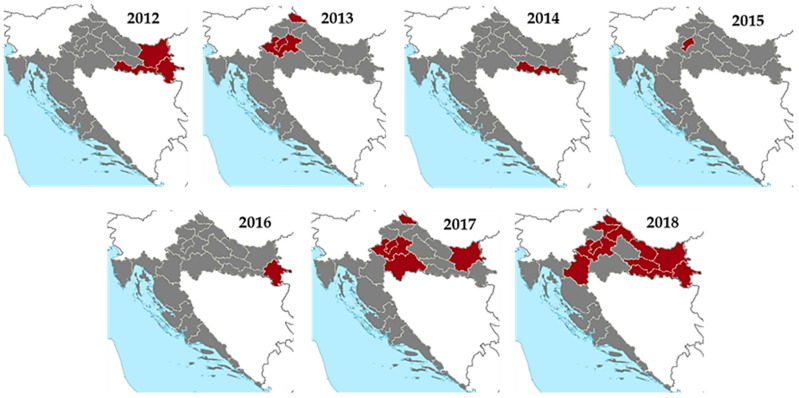
Geographic distribution of human WNV infections, 2012–2018.

WNV infections were reported from July to September, with a majority of patients detected in August (45.0%) and September (48.0%). The seasonal and geographic distribution of WNV cases are presented in Figure 1 and Figure 2. In both eastern and northwestern continental regions, a moderate positive correlation (r = 0.50 and 0.49, respectively) was determined between the number of human cases and average monthly air temperatures in 2018. Similarly, a weak positive correlation (r = 0.22 and 0.20, respectively) was demonstrated between the number of cases and temperature in previous transmission seasons (2012–2017). In contrast, the correlation between total monthly precipitation and the number of human WNV infections was weakly (eastern region; r = −0.13) to moderately negative (northwestern region; r = −0.56) in 2018. In previous WNV transmission seasons in Croatia, the correlation between the number of cases and precipitation was found to be weakly negative in the eastern region (r = −0.07) and weakly positive in the northwestern region (r = 0.09) [34].

The demographic and clinical characteristics of patients with WNV infection are presented in Table 2. The overall male to female ratio was 1.4:1. Patients with WNND were older (median age 64 years, IQR 57–76) than patients with WNV fever (median age 50 years, IQR 40–66). The majority of WNND (74.0%) occurred in the age groups of 50+ years.

The main clinical presentations of WNND in Croatian patients were meningitis/meningoencephalitis (96.8%), but some other rare manifestations were also reported. A fatal case of WNV encephalitis associated with an acute anteroseptal ST elevation myocardial infarction was described during the 2017 outbreak [38]. In 2018, retinitis caused by WNV was described in a patient with meningoencephalitis. The patient developed sudden decreased visual acuity in both eyes on the 9th day after disease onset. Fundus examination revealed vasculitis, edema, and hemorrhage of the entire retina in the posterior segment of the eye [39]. A combination of encephalitis and acute flaccid paresis, with cauda equina arachnoiditis as the main magnetic resonance finding, was another rare manifestation of WNV infection detected in a Croatian patient in 2018 [40]. Furthermore, WNV cerebellitis was documented in a 6-year-old child in 2018 [41].

The main cerebrospinal fluid (CSF) laboratory findings in patients with WNND included pleocytosis (median leukocytes 238, IQR 22–343) with mononuclear cells predominance (median 54%, IQR 30–75) and elevated protein levels (median 0.695 g/L, IQR 0.415–0.957).

Patients with both WNND and WNV fever showed a well-defined pattern of cytokine expression. In the CSF, increased concentrations of the key cytokines associated with innate and early acute phase responses (IL-6) and Th1 type immune responses (IFN-γ) were found. In contrast, expression of the key T-cell growth factor IL-2, Th17 cytokines, a Th2 cytokine IL-4 and the proinflammatory cytokine TNF-α appeared to be concentrated mainly in the periphery [42].

Twenty-three patients with severe WNND were hospitalized in the intensive care unit. The overall mortality in patients with WNND was 8.6%, while 21.7% of severe cases had moderate to severe disability at the end of the follow-up period.

Thus far, 100 laboratory confirmed clinical cases of WNV infections have been reported in Croatia, mainly WNND (*N* = 93). However, since the majority of infections are asymptomatic or presented as a mild flu-like disease (“summer flu”), the actual number of patients is probably underestimated.

## 3. WNV Infections in Horses

WNV NT antibodies were confirmed for the first time in Croatia in 4/980 (0.4%) randomly selected horses from eight Croatian counties tested between May 2001 and October 2002. No clinical signs were observed in seropositive horses at the time of sampling [43]. The geographic distribution and number of seropositive horses detected during 2010–2011 indicated that WNV was highly present in Croatia, spreading from east to west. WNV NT antibodies were detected in 3.43% (72/2098) of horses and 0.11% (3/2695) of cattle, with the highest seropositivity in eastern regions bordering Hungary and Serbia (horses 6.62–9.09%; cattle 0.36–0.65%). At the Croatian littoral, seropositive animals were found only at the westernmost Istria Penninsula (seroprevalence 2.78%), near the Italian and Slovenian borders. High seroprevalence rates detected in eastern regions suggested that eastern Croatia could be a part of larger WNV endemic area in Europe [9]. Results of the active WNV serosurveillance program performed during 2012 demonstrated asymptomatic acute infection in 12 sentinel horses (detection of IgM antibodies) before human cases were reported. Moreover, an increase in WNV IgG seropositivity in horses was observed in counties where human cases occurred (9.8% compared to 5.0% and 5.5% in 2010 and 2011, respectively), indicating an increased WNV circulation [32]. In 2013, 6.5% of horses tested positive for WNV IgG antibodies, while 0.3% showed serologic evidence of recent WNV infection. Seropositive animals were detected in 16/21 Croatian counties (all 14 continental counties and 2/7 counties located on the Adriatic Coast). The highest seroprevalence rate was reported in eastern and northwestern regions (up to 18.7 and 10.2%, respectively) [33]. WNV seropositive horses (seroprevalence 7.1–8.9%) were continuously recorded from 2014 to 2017 in continental and coastal Croatian counties. In addition, acute asymptomatic infections in sentinel horses were regularly reported. During the largest 2018 Croatian outbreak, the first IgM seropositive horse was detected in March; thereafter, IgM-positive animals were continuously reported until November. Acutely infected animals were recorded in seven counties, while IgG-positive animals were detected in all continental counties. WNV IgG seroprevalence rates varied from 3.2% to 26.0% with the highest seropositivity in regions with the largest number of human cases. In 2018, climate conditions in continental Croatia from April to October were very or extremely warm. During the whole year, the absolute air temperatures were above the multiannual average, which could explain an early start of the WNV transmission season [34]. High WNV seropositivity in horses was recorded in continental counties in 2019 and 2020, respectively (21.4% and 18.6%); however, no human clinical cases were reported. There was a significant positive correlation between human and horse seroprevalence rates (Spearman rank correlation coefficient rho = 0.774, *p* = 0.04).

**Figure 3:**
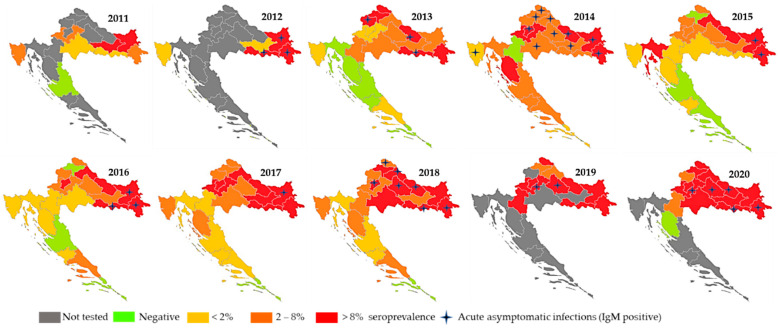
WNV infections in horses, 2011–2020.

The distribution of acute WNV infections and IgG seropositive horses is presented in Figure 3.

## 4. WNV Infections in Birds

**Figure 4:**
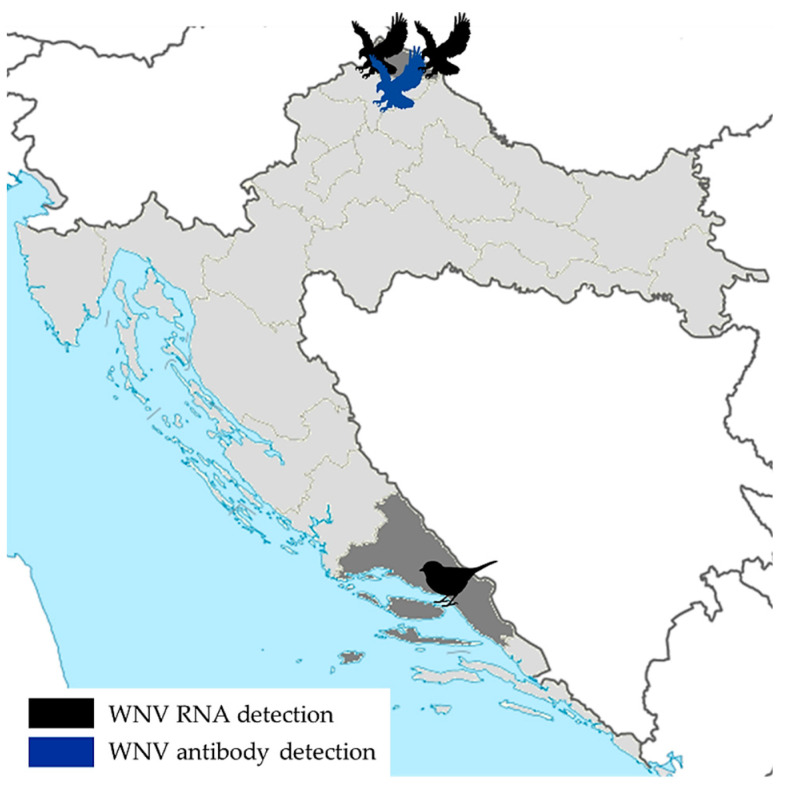
WNV infections in wild birds, 2018.

Although passive monitoring of flaviviruses in wild birds (virological testing of dead migratory birds, corvids and birds of prey) has been performed in Croatia since 2013, WNV was detected for the first time in two goshawks in the 2018 transmission season. During the 2018 outbreak, brain tissues of 35 dead wild birds from families Passeridae (*n* = 25), Accipitridae (*n* = 4), Laridae (*n* = 3), Turdidae (*n* = 1), Anatidae (*n* = 1) and Ciconiidae (*n* = 1) were tested for the presence of WNV RNA. In September 2018, a female and a male goshawk (*Accipiter gentilis*) from the same aviary in North-West Croatia were WNV RT-PCR-positive. In addition, WNV RNA was detected in one dead blackbird (*Turdus merula*) from a coastal Croatian county (Split–Dalmatia). Furthermore, WNV infection was serologically confirmed in one buzzard (*Buteo buteo*) with neurological symptoms from the same area where WNV-positive goshawks were detected [34] (Figure 4).

## 5. WNV Infections in Poultry

**Figure 5:**
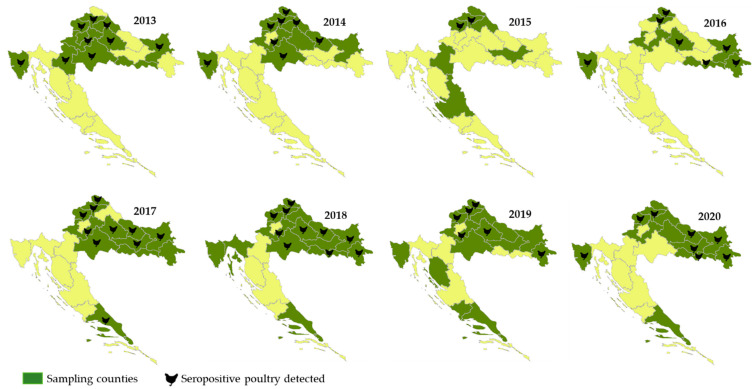
Geographic distribution of WNV seropositive poultry, 2013–2020.

The WNV activity in poultry was monitored using chickens (*Gallus gallus domesticus*) and turkeys (*Meleagris gallopavo domesticus*), since these species develop high titers of WNV-specific antibodies and low-level viremia following infection. The first serological evidence of WNV infection in poultry in Croatia was reported in 2013 in northwestern Croatia. During an eight-year period (2013–2020), serum samples from sentinel outdoor poultry were tested for the presence of WNV IgG antibodies. The poultry tested were older than three weeks and hatched in the current year or at the earliest in November/December in the previous year to assure a reliable demonstration of the viral activity in the current transmission season and the exclusion of maternally derived WNV antibodies as well as WNV antibodies from previous seasons. Serological surveys showed continuous WNV circulation in poultry with IgG seropositivity ranging from 1.8% to 22.9%. The majority of seropositive poultry was detected in continental Croatian counties (Figure 5) [35,44].

## 6. Mosquito Testing for WNV

Thus far, WNV was not detected in mosquitoes in Croatia. During the first WNV outbreak (September 2012), mosquitoes were sampled within the area of WNND occurrence in three northeastern counties. Medical entomological research was carried out in 64 localities; one of them was in the backyard of a WNND patient. A total of 1785 mosquitoes were captured. *Aedes vexans* species comprised 1634 specimens (91.54%), while 114 specimens (6.39%) were identified to be *Cx. pipiens* complex. All tested *Cx. pipiens* complex pools were negative for WNV RNA using a pan-flavivirus RT-PCR [45].

In the period from 2015 to 2020, an entomological survey for the presence of WNV in mosquitoes was performed in northwestern Croatia. It included two counties where WNV infections were detected in humans, horses, poultry and wild birds. A total of 618 mosquito sampling occasions were conducted, 613 in the Zagreb area and 5 in Međimurje County. In the Zagreb area, different areas were selected for mosquito sampling such as woods, populated areas close to the green belt, gardens in the urban part of the city, the city center close to Zagreb’s botanical garden, yards of citizens who complained of mosquitoes, open areas in public places (parks, cemeteries, green areas), walls in underground shelters, flats and cellars. Two mosquito samplings were in the backyard of WNND patients. In Međimurje County, mosquito samples were collected only once, in August 2017 at five locations. Four locations were placed next to chicken farms, and one was next to a horse stable. A total of 20,363 mosquitoes were collected and identified, of which 20,291 were from the city of Zagreb, and 72 mosquitoes were from Međimurje County. The mosquitoes belonged to 11 species. Of these, 31.1% of mosquitoes were identified as *Ochlerotatus sticticus*, 23.5% *Ae. albopictus*, 21.5% *Ae. vexans*, and 20.7% *Cx. pipiens* complex. Other species were represented by less than 2% [46,47]. None of the tested mosquito pool was positive for WNV RNA.

In addition, 2495 mosquitoes were collected for testing in Osijek–Baranja County (eastern Croatia) in 2019. Three species were detected: *Ae. vexans* (73.7%). *Cx. pipiens* (26.2%) and *Ae. albopictus* (0.1%). WNV RNA was not detected in any of the *Cx. pipiens* mosquito pools [48].

**Figure 6:**
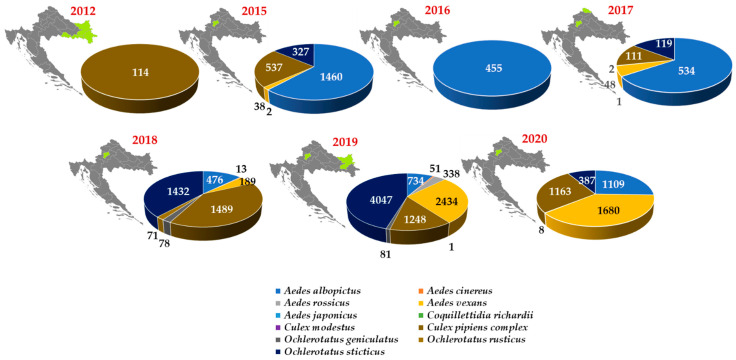
Mosquito sampling counties, number of mosquitoes and species tested for WNV in Croatia, 2012–2020 [43,45,46].

The mosquito sampling counties and number of mosquitoes tested for WNV are presented in Figure 6.

Even in the peak of outbreaks, it was hard to find positive mosquitoes. The best method was to sample mosquitoes around the houses with known WNV cases during the time of transmission. A relatively small number of collected mosquitoes may be the possible reason for negative results of WNV testing in mosquito pools [34].

## 7. Molecular Epidemiology of WNV in Croatia

During the first Croatian outbreak in 2012, WNV RNA was detected in two patients with WNND. Phylogenetic analyses of isolates based on a 167 bp fragment of the NS5 gene revealed WNV lineage 2. This represents the first molecular characterization of the circulating WNV strains in Croatia [49].

**Figure 7:**
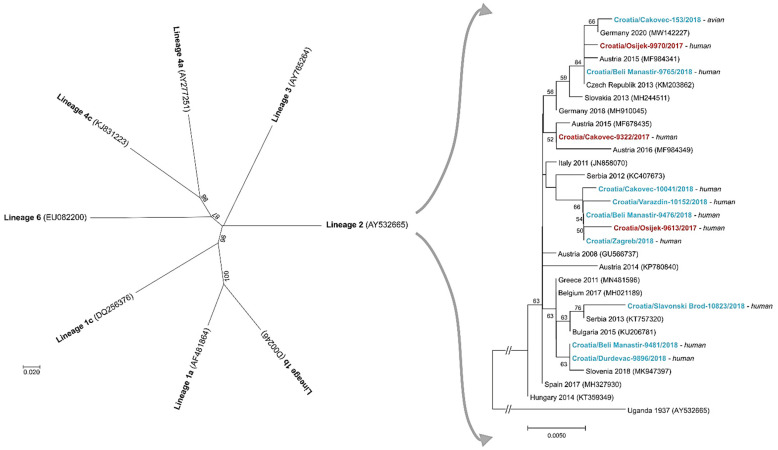
The rectangular phylogenetic tree displays genetic diversity of selected West Nile virus (WNV) isolates from Croatia, which belong to WNV lineage 2, as shown in the radial phylogenetic tree. The evolutionary history was inferred on 848 positions of the NS5 gene using the Neighbor-Joining method. Both trees are rooted with the first WNV strain (strain B956, Uganda 1937). Supporting (≥50%) bootstrap values of 1000 replicates are displayed at the nodes. Scale bars indicate nucleotide substitutions per site. The interrupted branches, indicated by double slashes, were shortened by 50% for better graphic representation. WNV isolates from Croatia are marked in bold and with hosts indicated next to the taxa. The Croatian isolates from 2017 are in red and isolates from 2018 in blue color. The other taxa are indicated by GenBank accession numbers as well as with the country of origin and isolation/detection year for those in the rectangular phylogenetic tree. The WNV lineages in the radial tree are proposed by Rizzoli et al. [22].

Thereafter, nucleotide sequences (an 848-nucleotide fragment of the WNV NS5 gene) were obtained for three and eight WNV strains detected in urine samples of patients with WNND in 2017 and 2018, respectively, as well as in a dead goshawk (*Accipiter gentilis*) in 2018. Phylogenetic analysis showed that all detected strains belong to WNV lineage 2 [34,50] (Figure 7).

## 8. Conclusions

After the first reported human clinical cases of WNV infection in 2012, sporadic cases as well as outbreaks (2012, 2013, 2017, 2018) were continuously recorded in continental Croatian counties. In addition, acute asymptomatic infections (IgM positive) as well as seropositive horses were regularly notified. Seroprevalence rates were higher in continental compared to coastal regions. In wild birds, WNV infections were detected during the large 2018 outbreak. WNV RNA was detected in two dead goshawks and one dead blackbird, while WNV antibodies were found in one buzzard with neurologic symptoms. WNV seropositive poultry were recorded each year from 2013 onward. Phylogenetic analysis showed that 11 strains detected in patients with WNND and one strain detected in a goshawk belonged to WNV lineage 2. Although mosquito samples were tested for the presence of WNV since 2012, none of the tested pool was positive for WNV RNA. Our results highlight the importance of a continuous interdisciplinary approach (‘One Health’) in WNV surveillance and prevention.

## Figures and Tables

**Table 1:** WNV infections in Croatia detected in humans and sentinel animals, 2011–2020.

Year	HUMANS		HORSES		BIRDS	POULTRY
	Acute Infections	Seroprevalence	Acute Infections	Seroprevalence	Virus Detection/Serologic Evidence *	Seroprevalence
2011	0	0.33%	NT	3.7%	NT	NT
2012	7	1.01%	12	8.7%	NT	NT
2013	20		9	6.5%	NT	22.9%
2014	1	NT	23	9.8%	NT	8.8%
2015	1	NT	10	7.1%	NT	3.1%
2016	2	NT	5	8.3%	NT	1.8%
2017	8	2.3%	1	8.9%	NT	10.3%
2018	61	2.9%	20	10.0%	*Accipiter gentilis* *Butteo butteo* *	3.5%
2019	0	1.6%	3	21.4%	NT	2.8%
2020	0	3.2%	14	18.6%	NT	5.8%

* serologic evidence; NT = not tested.

**Table 2:** Demographic and clinical characteristics of human WNV infections.

Characteristic	WNND; N (%)	WNF; N (%)
Gender		
Male	54 (58.1)	4 (57.1)
Female	39 (41.9)	3 (42.9)
Age group		
<30 years	4 (4.3)	0 (0)
30–39 years	4 (4.3)	1 (14.3)
40–49 years	11 (11.8)	0 (0)
50–59 years	25 (26.9)	4 (57.1)
60–69 years	9 (9.7)	2 (28.6)
70+ years	40 (43.0)	0 (0)
Clinical diagnosis (WNND)		NA
Meningitis	44 (47.4)	
Meningoencephalitis	34 (36.5)	
Meningitis/encephalitis, acute flaccid paralysis	12 (12.9)	
Other (cerebellitis, polyradiculitis, cauda equina arachnoiditis)	3 (3.2)	
TOTAL	93 (100%)	7 (100%)

WNND = West Nile neuroinvasive disease; WNF = West Nile fever; NA = not applicable.

## Data Availability

Not applicable.
